# Experimental study on the therapeutic effect and underlining mechanisms of positron in pancreatic cancer cells

**DOI:** 10.18632/oncotarget.18366

**Published:** 2017-06-05

**Authors:** Ying Wang, Ming Li, Rao Diao, Brian Tung, Dalong Zhang, Yaming Li

**Affiliations:** ^1^ Department of Nuclear Medicine, First Affiliated Hospital of China Medical University, Shenyang, Liaoning, China; ^2^ Department of Radiology, Massachusetts General Hospital, Boston, Massachusetts, USA; ^3^ Department of Urology, Shengjing Hospital of China Medical University, Shenyang, Liaoning, China

**Keywords:** pancreatic cancer, apoptosis, positron, ^18^F-FDG, microPET

## Abstract

The purpose of this study was to assess the potential therapeutic effect of positrons emitted by ^18^F-2-Deoxy-2-Fluoro-D-Glucose (^18^F-FDG) on pancreatic cancer cells and elucidate its underlying mechanisms. Pancreatic cancer cells were incubated with different radioactive concentrations of ^18^F-FDG and evaluated for anti-cancer properties and underlining mechanisms. In addition, three groups of tumor-bearing mice were treated with different doses of ^18^F-FDG weekly, the tumor growth rate was calculated, and the mice were imaged by positron emission tomography (PET) with ^18^F-FDG before and after treatment. The presence of apoptosis was detected by terminal deoxynucleotidyl transferase dUTP nick end labeling (TUNEL) stain and immunohistochemistry analysis. All treated groups exhibited positron-inhibited proliferation and positron-induced apoptosis compared with the control group *in vitro*. Further, we noted that higher treatment dose correlated with a better treatment response. *In vivo*, the high dose administration of ^18^F-FDG reduced tumor growth and prolonged the survival of treated mice compared with the control group with no change in the behavior or normal tissues of the mice. Immunohistochemical analysis and TUNEL stain showed more apoptotic cells than that in control group. The results demonstrated that positron radiation inhibited the proliferation and induced apoptosis of pancreatic cancer cells *in vitro* and *in vivo*, via an endogenous mitochondria-mediated signaling pathway.

## INTRODUCTION

Pancreatic cancer is the tenth most common malignant tumor worldwide with its incidence rising dramatically in recent years. It is highly lethal, and the prognosis remains poor. The most aggressive treatment regimen of surgical resection, chemotherapy and radiation are not effective [1–3], as shown by the dismal 5 years’ survival rate of 6% [4]. As a result, novel treatment modalities urgently need to be developed to combat this deadly cancer.

^18^F-FDG is widely used to evaluate malignant tumors in PET imaging [5]. It is an analogue of glucose that is taken up by the cells and phosphorylated by hexokinase into^18^F-FDG-6-phosphate. ^18^F-FDG-6-phosphate cannot be readily dephosphorylated, so it remains trapped in cells [6,7]. ^18^F emits positrons with an average energy of 0.250MeV and an abundance of 96%. Theoretically, a positron loses its kinetic energy and damages the tissue surrounded in the same manner as an electron [8]. Further, some research have confirmed that positron radiation has anti-proliferative efficacy [9,10]. In recent years, a number of studies [11–15] have shown the feasibility of using positron emitted by ^18^F-FDG to treat high glycolytic tumors, such as lung, breast, and colon cancer, by inducing apoptosis or necrosis and inhibiting tumor growth rate, definitively. Caridad et al [13] also found that positrons could inhibit the growth of lung metastases with no change in mice behavior or their normal tissues. The high glycolytic rate of pancreatic cancer cells reflects in the increased uptake and accumulation of ^18^F-FDG, making ^18^F-FDG a candidate agent for positron therapy against pancreatic cancers.

Herein, we identified the potential of ^18^F-FDG as a positron-emitting agent for the radiomolecular therapy of human pancreatic cancer *in vitro* and *in vivo* for the first time, and proposed a mechanistic rationale for the observed results. These findings may provide insight and serve as a foundation for the future application of ideal positron radionuclide as a novel treatment option for primary pancreatic cancer and its metastatic cancer.

## RESULTS

### Positron inhibits the proliferation of pancreatic cancer cells in a dose- and time- dependent manner

**Figure 1:**
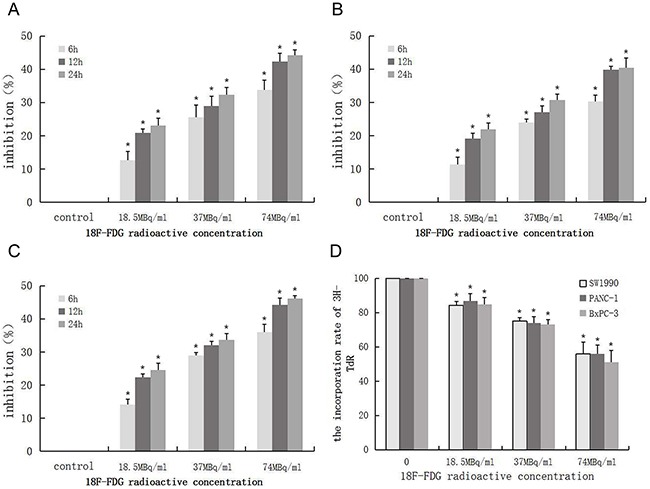
Positron inhibits the proliferation of pancreatic cancer cells in a time- and dose- dependent manner 3 pancreatic cancer cells were incubated with different concentrations of ^18^F-FDG for various time, the OD values was obtained through reading plate at 570nm with 96-well micro test spectrophotometer by MTT assay. The inhibition rate was expressed as the percentage of cell inhibition rate compared with the control group. The data were expressed as mean±SEM obtained from three samples. **(A)** The inhibition of ^18^F-FDG on SW1990 cell by MTT assay. **(B)** The inhibition of ^18^F-FDG on PANC-1 cell by MTT assay. **(C)** The inhibition of ^18^F-FDG on BxPC-3 cell by MTT assay. **(D)** SW1990, PANC-1, BxPC-3 cells were incubated with different concentrations of ^18^F-FDG for 24h, the CPM count of cell was obtained by the liquid scintillation counting method. The data were expressed as mean±SEM obtained from three samples. *p<0.01 versus control group.

To investigate the effect of positron on the proliferation of pancreatic cancer cells *in vitro*, we measured the growth of 3 pancreatic cancer cells (SW1990, PANC-1, BxPC-3) in different radioactive concentrations of ^18^F-FDG, using the MTT and ^3^H-TdR DNA synthesis assay. The MTT assay results showed that positron inhibited the proliferation in a time- and dose-dependent manner (Figure 1A–1C). The ^3^H-TdR DNA synthesis results showed that with the increase of ^18^F-FDG radioactive concentration, the counts per minute (CPM) of cells and incorporation rate of ^3^H-TdR gradually decreased compared with the control group (Figure 1D), which indicated that positron radiation could inhibit the DNA synthesis of pancreatic cancer cells.

### Positron induces apoptosis in pancreatic cancer cells

**Figure 2:**
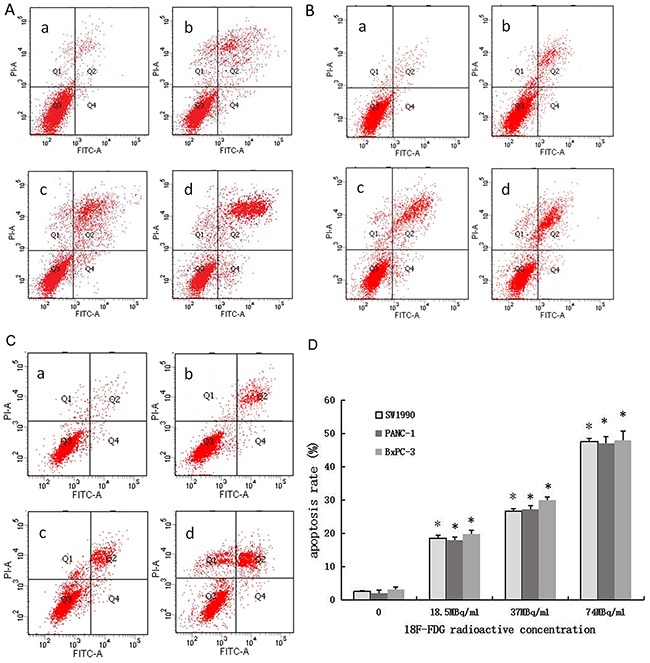
Positron induces apoptosis of pancreatic cancer cells in a dose-dependent manner 3 pancreatic cancer cells were incubated with different concentrations of ^18^F-FDG for 24h. **(A)** AnnexinV-FITC/PI staining of SW1990 cells treated by positron. **(B)** AnnexinV-FITC/PI staining of PANC-1 cells treated by positron. **(C)** AnnexinV-FITC/PI staining of BxPC-3 cells treated by positron. **(a)** control group; **(b)** 18.5MBq/ml ^18^F-FDG; **(c)** 37MBq/ml ^18^F-FDG; **(d)** 74MBq/ml ^18^F-FDG. **(D)** Statistical histogram. The data were expressed as mean±SEM obtained from three samples. *p<0.01 versus control group.

Pancreatic cancer cells (SW1990, PANC-1, BxPC-3) incubated with ^18^F-FDG for 24h displayed much higher rates of apoptosis than the control group (Figure 2A–2D). Most cells were in the late stages of apoptosis. With the increase of ^18^F-FDG radioactive concentration, the number of apoptotic cells accumulated, indicating that positrons may induce apoptosis in pancreatic cancer cells in a dose-dependent manner.

### Underlying mechanisms of apoptosis induced by positron in pancreatic cancer cells

**Figure 3:**
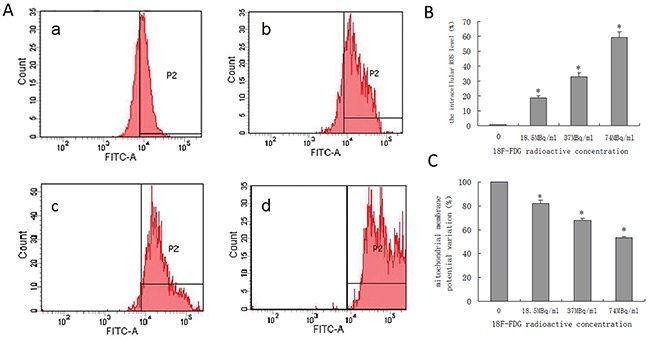
Quantified positron-induced the ROS generation and ΔΨm variation by flow cytometry in SW1990 pancreatic cancer cells Four groups cells were incubated with different concentrations ^18^F-FDG **(a)** control group; **(b)** 18.5MBq/ml; **(c)** 37MBq/ml; **(d)** 74MBq/ml. **(A)** Positron induced ROS generate in SW1990 pancreatic cancer cells in a dose-dependent manner. When incubated for 6h and harvested for FACS analysis. **(B)** statistical histogram of ROS. **(C)** Positron induced the ΔΨm of SW1990 cell decrease in a dose-dependent manner. When incubated for 6h and harvested for FACS analysis. The data were expressed as mean±SEM obtained from three independent samples. *p<0.01 versus control group.

We quantified the generation of reactive oxygen species (ROS) and the variation of mitochondrial membrane potential (ΔΨm) in SW1990 pancreatic cancer cells via flow cytometry, as shown in Figure 3A–3C. The results indicate that positron could induce the generation of intracellular ROS in the early period and decrease the ΔΨm in SW1990 pancreatic cancer cells in a dose-dependent manner.

**Figure 4:**
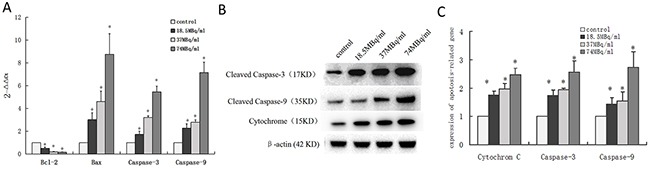
Underlying mechanisms of apoptosis induced by positron in SW1990 pancreatic cancer cells Four groups cells were incubated with different concentrations ^18^F-FDG **(a)** control group; **(b)** 18.5MBq/ml; **(c)** 37MBq/ml; **(d)** 74MBq/ml. **(A)** Real-time RT-PCR analysis for the mRNA expression of apotosis-related genes. Positron decreased Bcl-2 mRNA expression and increased Bax, Caspase-3, Caspase-9 and P53 mRNA expression in SW1990 cells in a dose-dependent manner. **(B)** Western blotting analysis for the Cleaved Caspase-3, Cytochrome C and Cleaved Caspase-9 protein treated with different concentrations ^18^F-FDG for 24h. **(C)** statistical histogram of western blotting. The data were expressed as mean±SEM obtained from three independent samples. *p<0.01 versus control group.

qRT-PCR analysis showed that mRNA expression level of Bcl-2 decreased and Bax, Caspase-3, and Caspase-9 increased in a dose-dependent manner after SW1990 pancreatic cancer cells were incubated with ^18^F-FDG for 24h (Figure 4A). These findings suggested positron-induced SW1990 cell apoptosis via regulating the expression of apoptosis-related genes.

To further confirm whether positron regulated the expression of apoptosis-related proteins in SW1990 pancreatic cancer cells, we performed western blot analysis after incubation with ^18^F-FDG for 24h. The proteins expression level of cleaved Caspase-3, Cytochrome C, and cleaved Caspase-9 increased in a dose-dependent manner (Figure 4B, 4C). This implicates the involvement of the mitochondrial pathways in positron-induced apoptosis.

### Positron inhibits tumor growth in tumor-bearing mice

**Figure 5:**
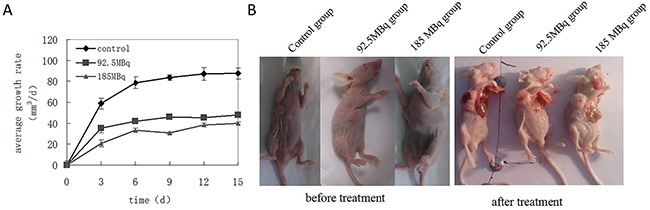
Positron inhibits the growth of tumor-burdened mice Eighteen tumor-burdened mice were randomly divided into 3 groups: control, 92.5MBq ^18^F-FDG and 185MBq ^18^F-FDG treatment groups. **(A)** Tumors were measured with the vernier caliper every 3 days, tumor average growth rates were monitored for 15 days after the experiment and compared with control group, the data were expressed as mean±SEM (n=6). It showed that the growth rate of the subcutaneously transplanted tumor slowed down gradually compared with control group (*p<0.05), especially in 185MBq ^18^F-FDG treatment group. **(B)** After experiment, all mice were killed, the tumor-burdened mice before and after the treatment, the tumor grew more slowly in treatment group compared with control group.

The tumor volume increased significantly over time, especially in the control group. 15 days into the experiment, three mice died in the control group, while the other three mice were noticeably thin, cachexic, and hypoergic. Transplanted tumors showed evidence of necrosis due to the significant enlargement of tumor size. In treatment groups, only one mouse in the lower dose ^18^F-FDG therapy group died. The growth rate of the subcutaneously transplanted tumors were drastically slowed compared with control group (Figure 5A), and the tumors grew more slowly in the treatment groups compared with the control group (Figure 5B).

### MicroPET imaging

**Figure 6:**
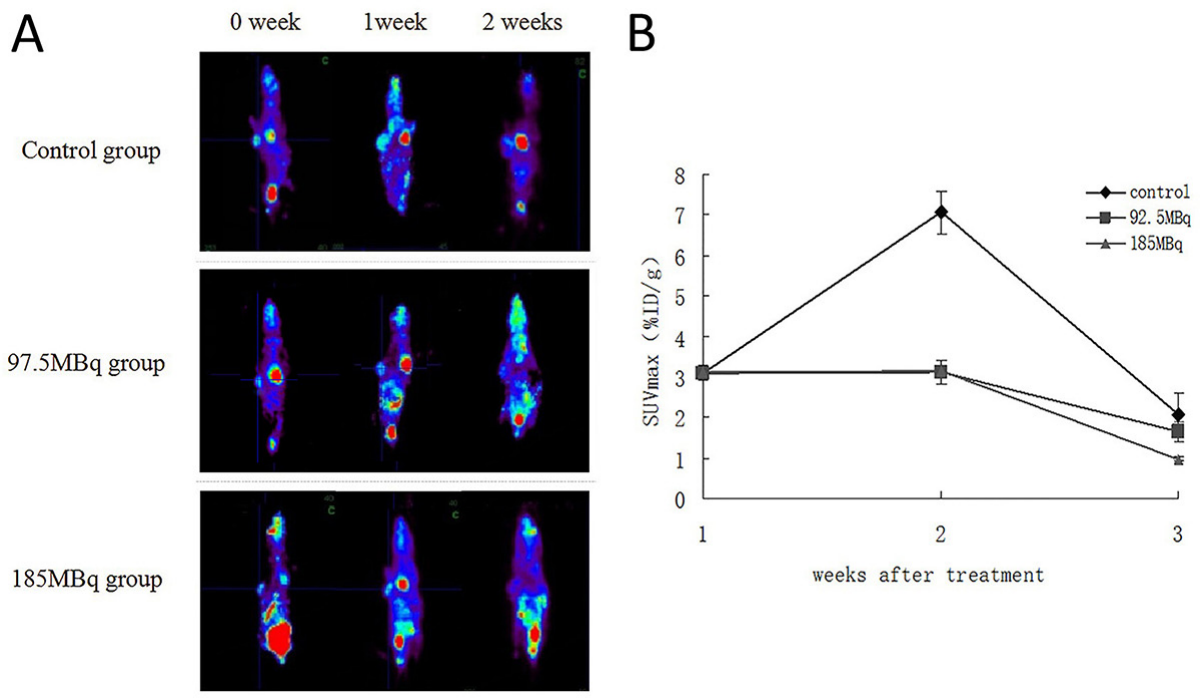
The baseline and follow-up microPET image of the tumor-burden mice in all groups Eighteen tumor-burdened mice were randomly divided into 3 groups: control, 92.5MBq ^18^F-FDG and 185MBq ^18^F-FDG treatment groups. **(A)** The baseline and follow-up microPET image of all groups. Animals were injected with 7.4MBq of ^18^F-FDG in caudal vein,anesthetized with isoflurane (0.3L/min, 1% in oxygen), and data were acquired for 50min. **(B)** Monitoring of tumor ^18^F-FDG uptakes before and after the treatment. The SUV_max_ were monitored for 2 weeks after treatment and compared with control group.

Subcutaneously transplanted tumors of all groups could be detected by microPET imaging. The SUV_max_ of tumors before treatment (week 0) was 3.09±0.14 (dose per gram of tumor, mean±standard deviation). The baseline and follow-up microPET images and quantitative analysis of tumors in all groups are shown in Figure 6A, 6B. In week 1, the tumor sizes in the control group increased significantly and were highly visible with increased ^18^F-FDG accumulation. In contrast, the tumor sizes in treatment groups increased at a moderate rate. The SUV_max_ of tumor in treatment groups were lower than control group in week 1, suggesting that positrons inhibited the growth of tumor to some extent. In week 2, the SUV_max_ of treatment groups was lower than that of week 1. However, because of significant enlargement of the tumor size in control group, the central necrosis was found, this may explain the lower radioactive nuclide concentration and lower SUV_max_ in control group. Thus, there were no significant differences in SUV_max_in tumors between the treatment groups and control group.

### Apoptosis of vital organs and tumors

**Figure 7:**
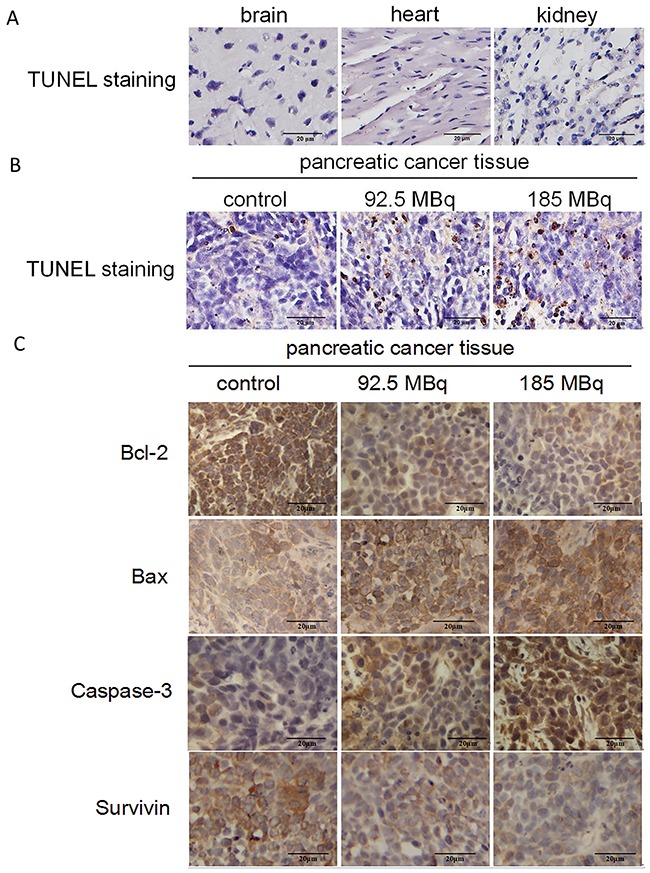
Tumor-burdened mice after treatment **(A)** TUNEL staining of main tissues after 185MBq ^18^F-FDG treatment. (Original magnification, 400×). **(B)** TUNEL staining of subcutaneous transplantation tumors after experiment, the tumors were obtained at 15 days after ^18^F-FDG treatment and compared with control group (*p<0.05). (Original magnification: 400×). **(C)** Immunohistochemical staining of bcl-2, bax, Caspase-3, Survivin of the subcutaneous transplantation tumors. The tumors were obtained and stained at 2 weeks after the experiment. The administered doses of ^18^F-FDG were 0, 92.5MBq and 185MBq, respectively. It showed that with the increase radioactive dose of ^18^F-FDG, expression level of Bcl-2 and Survivin reduced gradually, the expression level of Bax and Caspase-3 increased gradually compared with the control group (*p<0.05). (Original magnification, 400×).

The excretion of ^18^F-FDG is via the urinary system. There were nonspecific ^18^F-FDG accumulation in the urine and bladder and moderate ^18^F-FDG accumulation in the heart and brain tissues that may be subject to potential toxicity of positron therapy. The analysis of TUNEL staining showed no obviously apoptosis in these vital tissues in 185MBq ^18^F-FDG treatment groups (Figure 7A), indicating that a therapeutic dose of ^18^F-FDG had no conspicuous impairment on these organs. In contrast, apoptotic cells were observed in subcutaneously transplanted tumors in the treatment groups (Figure 7B). The higher the dose of ^18^F-FDG, the more positively-stained the cells were compared with control group.

### Immunohistochemical analysis of Bcl-2, Bax, Survivin and Caspase-3 expression

Immunohistochemical staining of Bcl-2, Bax, Survivin and Caspase-3 of tumor tissues in all groups are presented in Figure 7C. With the increase of ^18^F-FDG radioactive concentration, the expression level of Bcl-2 and Survivin decreased and Bax and Caspase-3 increased significantly compared with control group, indicating that therapeutic doses of positron could induce tumor cells apoptosis in a dose-dependent manner.

## DISCUSSION

The accumulation of ^18^F-FDG within the cells is in proportion to the cell's glycolytic rate [6,7]. The pancreatic cancer cells usually have high glycolytic rates, ^18^F-FDG accumulation within them is substantial, and thus the ‘metabolically trapped’ of ^18^F-FDG can deliver site-specific radioactivity to the tumors, and damage the tumor cells [8]. ^18^F emits positrons with a physical half-life of 109 minutes and an average energy of 0.250MeV and an abundance of 96%, which may be regarded to have limited tumoricidal effect. But in our study, we found its anti-cancer effect on pancreatic cancer cells *in vitro* and *in vivo*. Our data showed that positrons emitted by ^18^F-FDG inhibited proliferation and induced apoptosis in a dose-dependent and time-dependent manner.

The results of 3 pancreatic cancer cells treated with ^18^F-FDG demonstrated similar efficacy in inhibiting proliferation and inducing apoptosis, so we further investigated the underlying mechanisms of apoptosis induced by positron. ROS played an important role in apoptosis induced by positrons, we confirmed that the level of intracellular ROS rose significantly after the pancreatic cancer cells were incubated with ^18^F-FDG for 6 hours. The ionizing radiation of positron induced a variety of free radicals including ROS by the water radiolysis reaction, destroyed the molecular structure and promoted mitochondrial dysfunction. In theory, the abundant production of ROS can promote the excess accumulation of Ca^2+^, which may further damage mitochondria directly or indirectly by interrupting the function of the respiratory chain, subsequently leading to the loss of mitochondrial membrane potential, and eventually releasing cytochrome C. Cytochrome C binding with pro-Caspase-9 triggers the activation of the Caspase cascade and induces cell apoptosis [16–20]. In our present study, we confirmed the mitochondrial membrane potential decreased significantly and the protein expression levels of cytochrome C, cleaved Caspase-3, and cleaved Caspase-9 increased significantly in pancreatic cancer cells incubated with ^18^F-FDG for 24 hours.

In addition, apoptosis in pancreatic cancer cells induced by positrons was associated with the regulation of multiple genes. The intrinsic apoptosis pathway involves the Bcl-2 family [21–23], the members of which play an important role in apoptosis. Bcl-2 serves as the mitochondrial gate-keeper, which is usually over-expressed in tumors. At the same time, Bcl-2 promotes cell survival by limiting the pro-apoptotic effects of Bax and blocking the release of cytochrome C from mitochondria. The ratio of Bax to Bcl-2 is important for maintaining mitochondrial membrane integrity and influencing the initialization of apoptosis [24–26]. In our study, the qRT-PCR results showed that the expression level of Bcl-2 mRNA decreased and Bax mRNA increased significantly with higher incubated dose of ^18^F-FDG for 24 hours. The decrease of the Bcl-2/Bax ratio is associated with the initiation of cell apoptosis. Meanwhile, the release of cytochrome C from mitochondria further enhanced the activation of the Caspase cascade. We also found that the expression level of Caspase-3 and Caspase-9 mRNA increased at the same time, all of which indicated that the endogenous mitochondria- mediated signaling pathway plays an important role in positron-induced apoptosis of pancreatic cancer cells.

We further found that high doses of ^18^F-FDG significantly stagnated tumor growth in tumor-bearing mice. The tumor-bearing mice underwent the microPET scan weekly. The ^18^F-FDG uptake in treatment groups was lower than that of control group on week 1(*P*<0.05), which may demonstrate that positrons could inhibit the growth of tumors and dampen tumor cellular activity. Meanwhile, the SUV_max_ of treatment groups were notably lower on week 2 than on week 1, which indicated that repeated positron therapy may be useful.

Semi-quantitative analysis of TUNEL-positive cells demonstrated that there were more apoptotic cells in the tumors of the treatment groups than control group. Immuno histochemical analysis confirmed that the expression level of Bcl-2 and Survivin decreased significantly and the expression level of of Bax and Caspase-3 increased compared with the control group, which is similar to our findings *in vitro*, indicating that positrons could induce tumor cells apoptosis *in vivo*.

Some normal organs such as the brain and heart tissue showed higher ^18^F-FDG uptake. In addition, ^18^F-FDG is excreted via the kidney and bladder, all of which may be subjected to potential toxicity from positron therapy. Maybe they are relatively radioresistant, in our study we did not find conspicuous impairment in these organs. Moreover, Moadel RM et al [15] proposed some methods to minimize the damage to the kidney and bladder through diuretic administration and intermittent catheterization. Via phenobarbital or benzodiazepine therapy, we could reduce the uptake of the brain tissue. In addition, the cardiac uptake could be reduced by adhering to a low carbohydrate diet. Moreover, positron therapy doses could also be fractionated to avoid the damage to vital tissues. Jaini S et al [27] proposed that the use of various hormones such as estrogen, progesterone and oncogens to increase the expression of glucose transporters in cancer cells so as to potentiate the radiotoxicity of positron in tumor cells.

There are several possibilities of clinical application of targeted positron therapy in anti-tumor strategy. First of all, many positron emitters are halogens like ^18^F^-^. It is possible to incorporate them into small molecules which could deliver radioactivity deeply into the tumors in a homogeneous manner unlike radiolabeled antibodies which are traditionally used for the delivery of beta-emitters, as pancreatic tumors are very difficult to penetrate even with relatively small molecules. The high glycolytic rate of pancreatic cancer cells and some other cancer cells reflect in an increased uptake and accumulation of ^18^F-FDG. Secondly, the primary pancreatic cancer and its metastatic cancers have similar glycolytic rate or other metabolic characteristics, so it may treat the primary tumors and its metastases at the same time. Third, in our study, we did not find conspicuous impairment in the behavior of the mice and their normal organs, findings similar to many former studies, though these need to be further investigated. Furthermore, ^18^F possesses a shorter half-life and lower-energy positron compared to other positron-emitters. All of the studies about positron therapy showed the efficacy of ^18^F-FDG in tumors. We can use more ideal position radionuclide such as ^76^Br (half-life 16.2hours, 3.44MeV), ^124^I (4.2days, 2.13MeV), and ^64^Cu (12.7hours, 0.657MeV) [11], and develop targeted PET therapy based on metabolism, angiogenesis, receptor- mediated antibodies, or use other techniques to improve the efficacy of cancer positron- radiation. Paik JY et al [28] evaluated the combined treatment of nitric oxide stimulation with high dose ^18^F-FDG, which promoted apoptosis and enhanced positron radiation therapy to the actively proliferating angiogenic endothelial cells, which is mainly referred to tumor neovascularization. All of these could increase the potential usefulness of positron pharmaceuticals and make it a promising treatment option in the future.

In conclusion, this study demonstrates that positrons emitted by high dose ^18^F-FDG could inhibit proliferation and induce apoptosis in pancreatic cancer cells *in vitro* and *in vivo*. It appears that positrons induce apoptosis in pancreatic cancer cells by creating an ROS-rich milieu and activating endogenous mitochondria-mediated signaling pathway. This is seen in the decreased Bcl-2/Bax ratio and the elevated expression of cytochrome C, cleaved Caspase-3 and cleaved Caspase-9. The potential of other more ideal positron radionuclide in pancreatic cancer and its metastatic cancers therapeutics should be further studied.

## MATERIALS AND METHODS

### Cell lines, reagent and mice

The SW1990, PANC-1, BxPC-3 human pancreatic cancer cell lines were cultured in RPMI-1640 (Hyclone, Shanghai, China) supplemented with 10% fetal bovine serum (Hyclone, Shanghai, China) and 1% penicillin-streptomycin. The rabbit polyclonal antibodies specific for Bcl-2, Bax, Survivin, Caspase-3, Caspase-9 and Cytochome C were purchased from Boster (Wuhan, China). The MTT, ROS, Rhdomin123 reagent was purchased from Sigma, AnnexinV-FITC reagent was purchased from Biosea Biotechnology co, LTD (Beijing, China). The RNAiso Reagent kit for total RNA extraction and RT Reagent Kit for reverse transcription were obtained from Takara (Dalian, China). TUNEL apoptosis detection kit was purchased from keygen technology development co, LTD (Nanjing, China). Female BALB/c-nu mice were purchased from HFK Bio-Technology. co. LTD (Beijing, China) and maintained in the animal facility at China Medical University.

### Control groups and experimental groups *in vitro*

Brifely, pancreatic cancer cells were incubated with different radioactive concentrations of ^18^F-FDG (0, 18.5 MBq/ml, 37 MBq/ml, 74MBq/ml).

### Cell proliferation by MTT assay

The cell viability was determined by MTT assay. Briefly, the SW1990, PANC-1, BxPC-3 cells were dispensed in a 96-well culture plate at a density of 5×10^3^ cells per well and incubated at 37°C. After 24 hours of incubation, they were treated with different radioactive concentrations of ^18^F-FDG (0, 18.5MBq/ml, 37MBq /ml, 74MBq/ml) for 6h, 12h and 24h. Following treatment, the cells were further incubated with 20uL of MTT reagents (5mg/mL) for 4 hours at 37°C before DMSO was added, to dissolve the formazan crystals. The absorbance was measured at 570 nm in the ultraviolet spectrophotometry (MULTISKAN GO, Thermo).

### DNA synthesis analysis

The SW1990, PANC-1, BxPC-3 cells were incubated with different radioactive concentration^18^F-FDG (0, 18.5MBq/ml, 37MBq/ml, 74MBq/ml) for 24 hours, incubated cells (1×10^6^) of each group with 1.5μl ^3^H-TdR each wells for another 24h, then put them into the scintillation liquids and incubated for 24 hours at 37°C, using the liquid scintillation counting method to detect the CPM of the cell and analysed DNA synthesis in the cell.

### Annexin V/PI staining assay for apoptosis

The SW1990, PANC-1, BxPC-3 cells (1×10^6^) from each group at 24h were collected and washed with cold PBS (PH 7.4). According to the reagent, add the buffer, Annexin V and PI, the apoptotic peak in each group was detected by flow cytometry. The assay was repeated three times.

### Measurement of reactive oxygen species (ROS)

The intracellular changes in ROS generation were detected by staining the cells with 2,7- dichlorodihydrofluoresceindiacetate (DCFH-DA). Briefly, SW1990 cells (1×10^6^) were incubated with different radioactive concentrations of ^18^F-FDG (0, 18.5 MBq/ml, 37 MBq/ml, 74MBq/ml) for 6h. After that the cells were incubated with 20μm DCFH-DA for 20min and washed with cold PBS (PH 7.4). Subsequently cells of different groups were harvested and resuspended in PBS before the fluorescence was analysed by flow cytometry.

### Measurement of mitochondrial membrane potential (ΔΨm)

Fluorochrome dye Rhdomin123 was used to evaluate the changes in ΔΨm. Brifely, SW1990 cells (1×10^6^) were incubated with different radioactive concentrations of ^18^F-FDG (0, 18.5 MBq/ml, 37MBq/ml, 74MBq/ml) for 24h. After that the cells were incubated with Rhdomin123 for 30min and washed with cold PBS (PH 7.4). Subsequently cells of different groups were harvested and resuspended in PBS before the fluorescence was analysed by flow cytometry and observed by the fluorescence microscope the change of mitochondria membrane potential.

### Real-time quantitative reverse transcription (qRT)-PCR and western blot analysis

Incubated SW1990 pancreatic cancer cell (2×10^6^) with different radioactive concentrations of ^18^F-FDG (0, 18.5 MBq/ml, 37 MBq /ml, 74MBq/ml) for 24h. Briefly, the total cellular RNA of each group were extracted with Trizol. After that using the reagent to reversely transcribed the first-strand cDNA. PCR was carried out using cDNA as the template and the following primers:F:TGTGGCATTGAGACAGAC,R: CATGGCACAAAGCGACTG for Caspase-3. F: TCCT GGCAAAAGGTCAGAGT, R: GTTGTGTGTTCGCC TCTTGA for Cytochrome c. F: GCGAACTAACAGGCA AGCAGCAA, R: CTCAAGAGCACCGACATCACCAAA for Caspase-9. F: CAATGACCCCTTCATTGACC, R: GATCTCGCTCCT GGAAGATG for human GAPDH. The analyses of PCR result was performed on ΔΔCt method [29]. For western blot analysis, cells from different experimental groups were collected, washed with cold PBS and lysed. Total cellular lysates were then resolved by 12% sulfate-polyacrylamide gel electrophoresis and subsequently transferred onto polyvinylid- enedifluoride membranes. The membrane was then blocked with 5% non-fatdry milk in Tris-buffered saline containing 0.05% Tween-20, incubated with rabbit polyclonal antibodies specific for Caspase-3, Caspase-9, Cytochome C and β-actin (an internal control) in the blocking solutionfor one night and then incubated with peroxidase (HRP)-conjugated secondary antibody for additional 2h. Subsequently, the membrane was washed and developed using the Super-Enhanced chemiluminescence detection kit according to the manufacturer's instructions. The protein bands were visualized after exposure of the membranes to X-ray film.

### Animal model

All procedures involving mice were conducted according to the guidelines of the Institutional Animal Care and Use Committee of China Medical University. Four- to five-week old female nude mice were injected subcutaneously on the armpit area of their right anterior limbs with 100ul cell suspension containing 2×10^6^ SW1990 cells. The ninth day after implantation the tumor reached 0.7 cm in diameter and the imaging and therapy with ^18^F-FDG was initiated.

### PET/CT image of tumor-bearing mice with ^18^F-FDG

Tumor-bearing pancreatic cancer mice models were fasted for at least 8 hours before injecting 7.4MBq ^18^F-FDG in caudal vein. The mice rested about 50 minutes for the uptake of ^18^F-FDG under anesthesia. The PET scan was done on a microPET scanner (Metis Small Animal PET, Shandong Madic Technology Co, Ltd), after 20 minutes emission scan the ^18^F-FDG microPET image of the tumor-bearing mice was obtained. The SUV_max_ of the tumor was calculated in the PET image.

### Treatment of tumor-bearing mice with ^18^F-FDG

Eighteen tumor-bearing mice with tumor sizes of 0.70-0.90cm in diameter were randomly divided into 3 groups (control group and two treatment groups). The two treatment groups were separately injected with a dose of 92.5MBq and 185MBq ^18^F-FDG in the caudal vein on a weekly basis. The therapeutic doses were based on previously published research (12,14-16). The control group was injected with saline, after having fasted for at least 8 hours. All mice rested for the uptake period of 50 minutes under anesthesia. Mice were monitored until half of the control group were dead. We acquired the microPET image and measured the SUV_max_ of the tumor before and after treatment. Animals were anesthetized with isoflurane (0.3L/min, 1% in oxygen), then injected with 7.4MBq of ^18^F-FDG in the caudal vein and allowed to circulate for 50 minutes prior to image acquisition. Tumors were measured with the vernier caliper every 3 days, the volume of the tumors was calculated according to the following formula: V = ab^2^×0.52 (a is the larger and b is the smaller of the two dimensions). The tumor growth rate (cm3/day) was calculated according to the following formula: growth rate = (tumor volume_initial_ - tumor volume_N day after treatment_) / N ×100%.

### Immunohistochemical analysis of Bcl-2, Bax, Survivin and Caspase-3 expression

After experiment, all mice were sacrificed. The tumor tissues were paraformaldehyde- fixed, paraffin-embedded, cut into serial sections (5μm) and then mounted on glass slides and dried at 60°C for 2 h, tumor sections were consecutively incubated: 15 minutes in PBST (phosphate-buffered saline with 0.3% TritonX-100), 30 minutes in blocking buffer (PBST, 5% normal goat serum, 0.2% bovine serum albumin), in Rabbit polyclonal Bcl-2, Bax, Survivin and Caspase-3 isoform-specific antibodier in blocking buffer for an hour, 15 minutes in PBST, an hour in rabbit anti-goat IgG (conjugated with fluorescein) in blocking buffer and 30minutes in PBST.

### Apoptosis detection

TUNEL assay was used to detect apoptosis of the resected tumors and normal organs. The apoptosis of positive cells in five random viewpoints (×400) were counted, and then the mean and standard deviation were calculated for statistical analysis.

### Statistical analysis

Results were expressed as the means ± SEM. The statistical significance of differences in groups were evaluated by chi-square test and analysis of variance (ANOVA). All statistical analysis were performed using the SPSS 17.0 software. A value of *p* < 0.05 was considered statistical significance.

## Abbreviations

^18^F-FDG: ^18^F-2-Deoxy-2-Fluoro-D-Glucose
PET: positron emission tomography
SUV: standard uptake value
ROS: reactive oxygen species
PBS: phosphate buffered solution
PI: propidium iodide
MTT: 3-[45-dimethylthiazol-z-yl]-,5-DIPhengltetrazoliumbromide
DMSO: dimethyl sulfoxide
CPM: counts per minute
qRT-PCR: quantitative reverse transcription polymerase chain reaction
Caspase: cysteine proteinases with specificity
TUNEL: terminal deoxynucleotidyl transferase dUTP nick end labeling.
